# Surgical Reconstruction of the Urinary Sphincter after Traumatic Longitudinal Disruption

**DOI:** 10.1155/2014/176073

**Published:** 2014-09-02

**Authors:** Peter Rehder, Florian Schillfahrt, Viktor Skradski

**Affiliations:** Department of Urology, Medical University Innsbruck, 35 Anich Street, 6020 Innsbruck, Austria

## Abstract

The question is whether the urethral sphincter may be reconstructed after longitudinal injury similar to anal sphincter injuries. Analogue to obstetric, anal sphincter repair, an approximation repair of the sphincter may be feasible. An overlap repair is possible in anal sphincter repair, but because of the little tissue available in the urethral sphincter this is not an option. We describe three cases of urethral sphincter injury of different aetiologies. All resulted in a total longitudinal disruption of the muscular components of the urethral sphincter complex. After making the diagnosis of urethral sphincter injury, a primary approximation repair was done. Follow-up of at least two and up to three years is promising with one male patient being completely continent and the two female patients needing one safety pad per day. Longitudinal disruption of the muscular elements of the sphincteric urethra may be primarily reconstructed with good success using an approximation technique with simple interrupted sutures.

## 1. Introduction

Trauma to the urinary sphincter mechanism might lead to severe urinary incontinence and/or urethral stricture formation. Injury to the muscle components of the urinary sphincter may be the result of trauma during birth, as part of blunt or penetrating trauma to the pelvis or as the result of surgery. We know from obstetric experience that the torn or ruptured anal sphincter may be surgically reconstructed with good results [1, 2]. The urinary sphincter is rather well protected underneath the pubic symphysis suspended within the pelvic floor. Childbirth may cause postpartum urinary incontinence although the exact mechanism of injury is poorly understood. Episiotomy does not seem to have a protective effect [3]. Longitudinal tears into the sphincter mechanism from the bladder wall through the bladder neck may accompany pelvic fracture injuries in persons with full bladders at the time of impact. Severe or direct trauma to the sphincteric urethra may lead to complete disruption of all muscular elements. Transection of the membranous urethra may be dealt with by primary realignment or suprapubic cystostomy alone and delayed repair [4, 5]. Is it then possible to reconstruct the sphincteric urethra when all muscular elements have been cut in a longitudinal fashion? Included in this case series are patients with direct injury to the whole length of the sphincteric urethra, where the urethral lesion is limited to a longitudinal cut through all muscular elements of the sphincter complex.

## 2. Materials and Methods

This case series includes three patients of which each had a total longitudinal disruption of all muscular elements of the urethral sphincter. The diagnosis of longitudinal disruption of the urethral sphincter was made followed by primary reconstruction with interrupted sutures using an approximation technique. The muscular elements were approximated taking care not to strangulate the tissues by over tightening the knots. A simple suture technique was used rather than mattress sutures as in the case of anal sphincter repair.

### 2.1. Case  1: Urethral Sphincter Injury due to Blunt Pelvic Trauma

**Figure 1:**
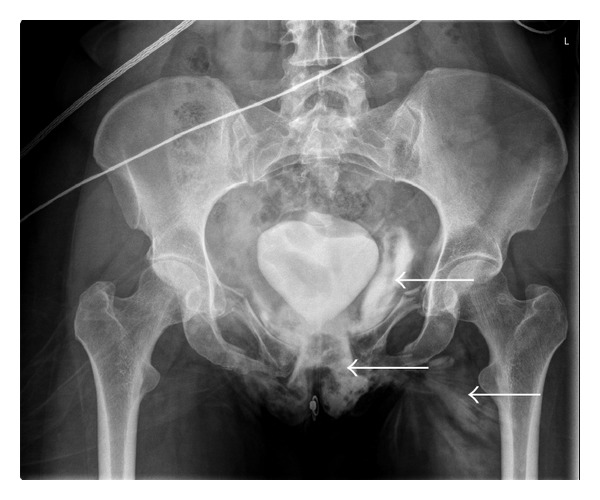
Severe extravasation of contrast solution into the soft tissues is shown (white arrows), yet demonstrating an intact bladder. In the presence of a longitudinal urethral injury contrast solution runs freely into the surrounding soft tissues. The bladder is “pushed up” by a large pelvic haematoma.

**Figure 2:**
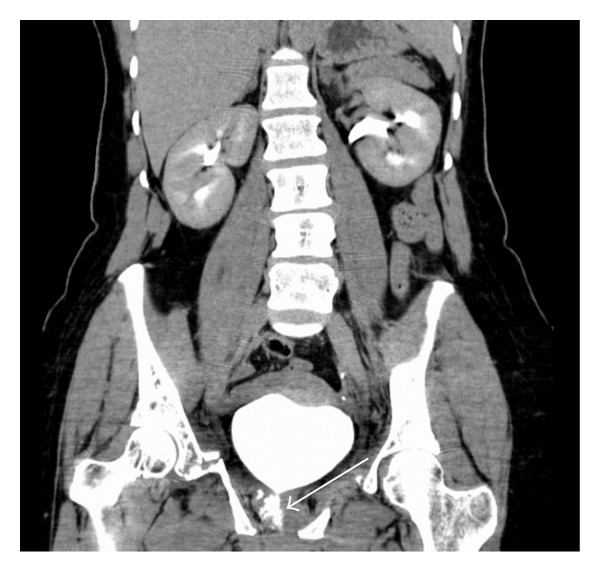
CT scan (early excretory phase) showing leakage of contrast solution from the proximal urethra into the surrounding tissues (white arrow). This typifies a urethral injury with the intact bladder.

**Figure 3:**
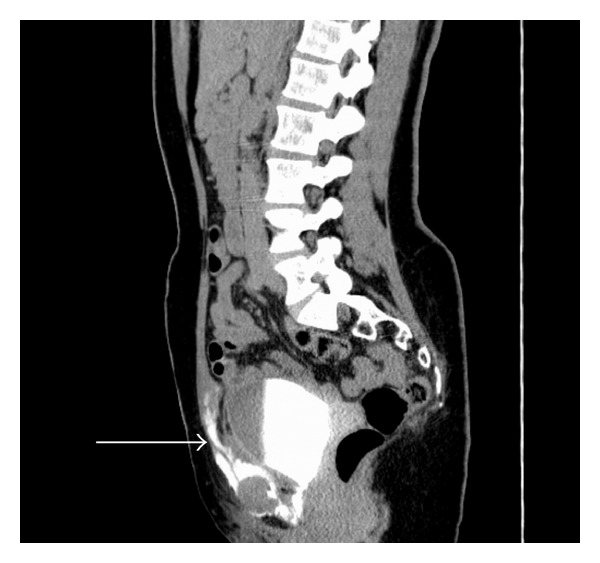
Lateral view showing extravasation of contrast solution into the soft tissues of the lower abdomen (white arrow).

A 49-year-old woman had an accident while riding her motorbike. She crashed straight into an oncoming motor vehicle fracturing her pelvis. On presentation she had genitoperineal pain with severely swollen labia and tenderness along the left lower abdomen. The patient was stabilized and a whole body CT scan was performed. Bilateral fractures of both superior and inferior pubic rami as well as diastasis of the pubic symphysis were diagnosed. Both kidneys and the bladder were normal. During the excretory phase, severe extravasation into the genitoperineal area and lower abdominal wall was noted (Figure 1). A clear urethral contour could not be seen. Local examination of the external genitalia was limited due to severe swelling of both labia majora. A transurethral catheter was successfully passed draining macroscopically clear urine from the bladder. The clinical diagnosis of a urethral rupture was made (Figures 2 and 3). It was decided to do an immediate extra peritoneal exploration of the bladder and urethra followed by orthopaedic fixation of the pelvic fracture. The urethra was totally open from bladder neck to meatus with the catheter inside. The edges of the urethral mucosa and sphincter could be clearly identified. Six single simple interrupted through-and-through sutures were placed into the muscular aspects of the urinary sphincter. The first sutures were not tied to function as stay sutures to simplify the placement of the following sutures. After placement of all sutures they were loosely tied so as to accurately approximate the edges. Care was taken not to strangulate the tissues. The transurethral catheter was kept for three weeks and removed at the time of a micturition cystogram.

### 2.2. Case  2: Urethral Sphincter Injury due to Perineal Impalement

**Figure 4:**
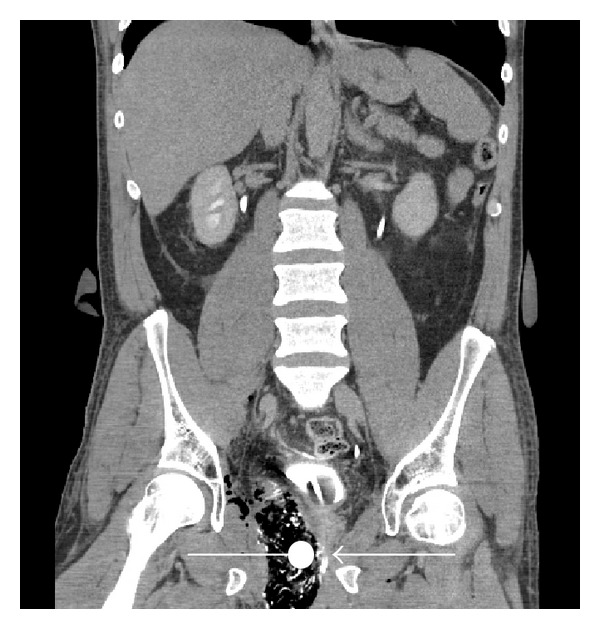
CT scan showing a big swab from the perineum into small pelvis to the right. Note that the swab (round mark) lies directly adjacent to the membranous urethra/transurethral catheter (arrow).

**Figure 5:**
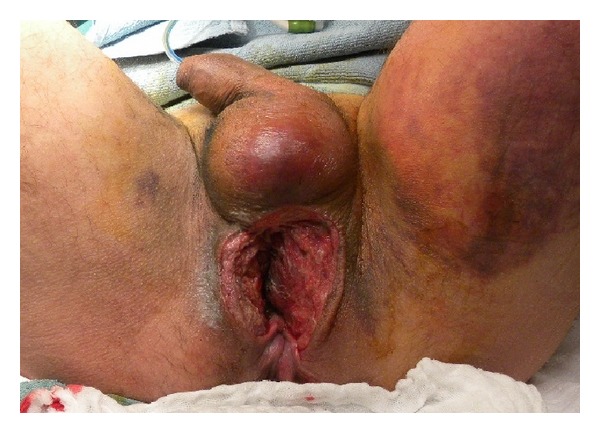
The big perineal defect and the extent of the anal sphincter injury are clear. The anal mucosa has already been closed at the time of initial debridement.

**Figure 6:**
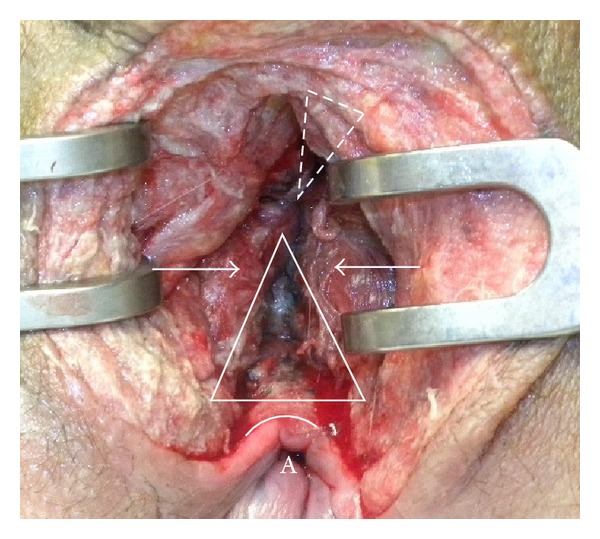
The defect in ventral anal sphincter (white triangle) is demonstrated. The muscular aspects of the anal sphincter are indicated with white arrows. Not clearly shown is the defect in the dorsal urethral sphincter complex (upside down dotted triangle) as it lies deeper. At this stage the anal mucosa (white arch) was already closed. A = anus.

A 42-year-old male fell during work on a fence resulting in a severe perineal impalement injury leading to life threatening bleeding and an obvious anal injury. A midline lower abdominal incision was made. No intraperitoneal injuries could be seen, and a defunctioning colostomy in the left lower abdomen was done. The perineum was debrided and the open anal mucosa sutured. At this stage no attempt was made to reconstruct the anal sphincter mechanism. The right internal iliac artery was ligated, but the bleeding could only be controlled by packing the small pelvis with large swabs. This was done through the open perineum. After patient stabilization, a helicopter transfer was organized to a tertiary trauma centre. The CT scan at that stage showed isolated extra peritoneal soft tissue injury from the perineum extending into the left small pelvis (Figure 4). No active bleeding, bowel, or bladder injury could be determined. Clinical examination confirmed an open book fracture of the pelvis, total ventral full thickness disruption of the anal sphincter complex, and total disruption of the dorsal urinary sphincter complex. There was a big defect in the perineum, demonstrating the extent of the impalement injury (Figures 5 and 6). Both the anal and urethral sphincters were repaired using an approximation technique. Mattress sutures were used for the anal sphincter and simple sutures were used for the urethral sphincter. The corpora cavernosa that were ripped far apart were also approximated ventral to the sphincteric urethra. After sphincter repair, a vacuum device was placed until it was safe to perform secondary wound closure. The transurethral catheter was left in position for three weeks. A micturating cystourethrogram was done at the time of catheter removal and no extravasation was noted. At this time, the patient was still incontinent of urine and a transurethral catheter was placed for another 6 weeks until the patient was mobilized. The patient became continent after pelvic floor exercises accompanied by a pelvic floor-trained physiotherapist after six months.

### 2.3. Case  3: Urethral Sphincter Injury due to Urethral Diverticulum Surgery

**Figure 7:**
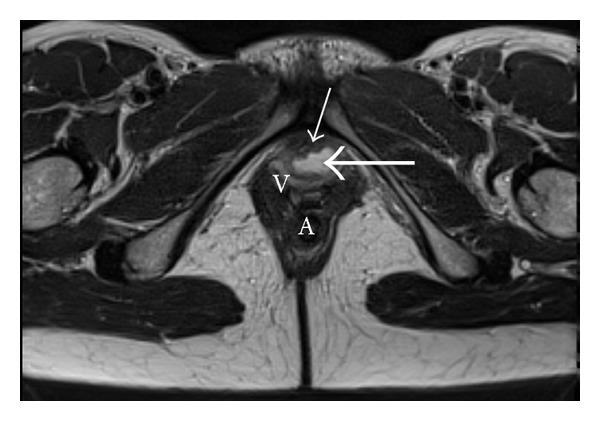
The dorsal urethral diverticulum (long white arrow), adjacent to the urethral lumen (short white arrow), as seen on MR examination. V = vagina; A = anus.

**Figure 8:**
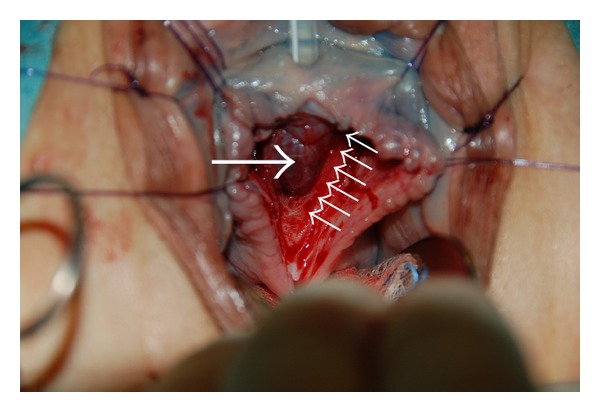
The urethral mucosa (long white arrow) is visible after excision of the urethral diverticulum. The dorsal muscular components (small white arrows) of the urethral sphincter complex are completely cut longitudinally thereby exposing the urethral mucosa from bladder neck up to the meatus.

**Figure 9:**
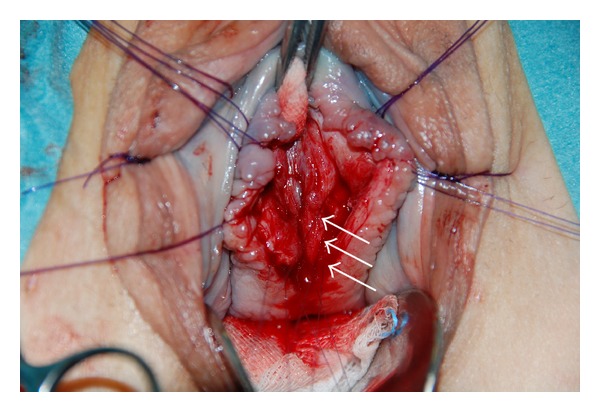
Four thin synthetic monofilament absorbable sutures (three sutures indicated with white arrows) are used to approximate the muscular components of the urethral sphincter. A total of eight sutures were placed to approximate the sphincter edges/fascia surrounding the sphincter.

A 49-year-old woman presented with a painful periurethral mass. Puss drained spontaneously after which the patient had a MRI of the small pelvis (Figure 7). This confirmed a large periurethral diverticulum dorsal to the urethra penetrating the urethral sphincter. The diverticulum measured 4 × 3 × 3 cm and was symptomatic in the sense that the patient felt an anterior vaginal mass which also interfered with sexual intercourse. After an informed consent was obtained, it was decided to excise the urethral diverticulum. During the exploration through an anterior midline colpotomy the diverticulum was identified. Dissection followed alongside the diverticulum up to the point where it entered the urethral lumen. During the dissection all dorsal midline muscular elements of the sphincter complex were opened until there was only urothelial mucosa left lining the urethral lumen from the bladder neck up to the urethral meatus (Figure 8). The muscular sphincter elements were approximated by thin simple interrupted sutures, after which the colpotomy was closed in layers (Figure 9). The transurethral catheter was kept for three weeks.

### 2.4. Surgical Technique

First of all the diagnosis of a urethral sphincter injury should be positively made. The extent of injury and all anatomic landmarks should be identified. Simple transection injuries may be dealt with by carefully placed interrupted simple sutures. When there is discontinuity of the sphincter circumference by a total longitudinal tear this should be specifically noted. The identification of the muscle components of the sphincter complex is important to be able to accurately place single thin monofilament sutures through all layers. The sphincteric urethra may be three to five cm long, and the sutures are only tied after all sutures have been placed. The idea is to approximate the muscular sphincter components loosely taking care not to constrict the blood supply of the sphincter itself. By using thin monofilament resorbable suture material postoperative scarring is minimized. Care should be taken to tie the sutures free of tension and torsion [6]. Polyfilament sutures might cause additional shear trauma to the muscle elements. All in all it boils down to simple accurate approximation to realign those tissues that belong together. The urethral sphincter repair should be analogue to the approximation repair in obstetric anal sphincter repair.

## 3. Results and Discussion

Two years after urethral trauma and reconstruction the first patient is socially continent. During maximal straining she loses a few drops of urine and uses one safety pad per day. The second patient after three years of follow-up is totally continent of urine. After the colostomy was reversed he suffers from occasional stool smearing. The fact that he is continent of urine might relate to an intact bladder neck. He is able to interrupt his stream during voiding. The third patient is socially continent needing no pad per day. When her bladder is full and she has an urge to void, she might lose a few drops of urine. These patients demonstrate that it is possible to reconstruct the urethral sphincter using an approximation technique.

A Young-Dees-Leadbetter procedure seems to help regain continence after pelvic fracture injuries in children [7]. In cases of membranous urethral rupture with secondary stricture formation, the urethroplasty focuses on regaining a patent urethral lumen rather than trying to reconstruct the sphincter mechanism. Often the membranous urethra is torn off the bulbous urethra during pelvic trauma sparing sphincteric function [8]. The presence of an intact bladder neck often guarantees continence despite an incompetent urethral sphincter. The urethral sphincter complex has been reconstructed with varied success or failure in cases of bladder exstrophy. Gupta describes an anatomic repair of the bladder neck trying to maintain the innervation to the sphincter complex during sphincteroplasty.

Acute posttraumatic sphincter repair after longitudinal cuts through the whole length of the urethral sphincter has not been specifically described in the literature.

## 4. Conclusions

Longitudinal urethral injury to the sphincter complex is rare. In most cases of pelvic fracture injuries including various lesions to the urethra, the placement of a transurethral catheter will allow satisfactory healing of the sphincter mechanism. When the whole sphincteric urethral length is involved, it means that the urine diffuses freely into all surrounding tissues. This will mostly result in severe surrounding fibrosis and urethral stricture formation. Obvious severe urethral lesions should be recognized, and longitudinal tears diagnosed. This case series shows that an approximation technique of primary urethral sphincter repair may be successful. This may help to minimize future urethral stricture formation and may even result in acceptable continence rates in these patients. When sphincter structures are positively identified, a primary repair should be attempted.
